# Sugar Sweetened Beverage Consumption among Adults with Gout or Type 2 Diabetes

**DOI:** 10.1371/journal.pone.0125543

**Published:** 2015-05-15

**Authors:** Rinki Murphy, Simon Thornley, Janak de Zoysa, Lisa K. Stamp, Nicola Dalbeth, Tony R. Merriman

**Affiliations:** 1 Department of Medicine, Faculty of Medical and Health Sciences, University of Auckland, Auckland, New Zealand; 2 Maurice Wilkins Centre for Biodiscovery, Auckland, New Zealand; 3 Population Health Directorate, Counties Manukau District Health Board, Auckland, New Zealand; 4 Department of Renal Medicine, Waitemata District Health Board, Auckland, New Zealand; 5 Department of Medicine, University of Otago, Christchurch, New Zealand; 6 Department of Biochemistry, University of Otago, Dunedin, New Zealand; Uppsala Clinical Research Center, SWEDEN

## Abstract

**Background:**

Current guidelines for the management of type 2 diabetes and gout recommend that people with these conditions limit their sugar sweetened beverage (SSB) intake. We examined self-reported SSB intake among New Zealand adults with gout or type 2 diabetes, including those on hemodialysis.

**Method:**

1023 adults with gout and 580 adults (including 206 receiving hemodialysis) with type 2 diabetes, participated in this study of between 2009 and 2012. Participants completed an interviewer-administered SSB intake question “how many sugar sweetened drinks (including fruit juice), but not including diet drinks, do you normally drink per day?” SSB consumption was recorded as a circled number 0, 1, 2, 3, 4, 5, or >5, cans or large glasses (300mL) per day.

**Results:**

Consuming one or more SSB per day was reported by 64% (622/1023) of subjects with gout, 49% (176/374) with type 2 diabetes without dialysis, and 47% (96/206) with diabetes on dialysis. Consuming four or more SSBs per day was reported by 18% (179/1023), 9% (31/374) and 9% (18/206), respectively. Such high consumers of SSB were characterized after multivariable analysis to be more likely to be male (adjusted odds ratio (OR) 1.8; 95% confidence interval 1.1–2.9), younger in age (40 vs 65 years: 1.6; 1.1–2.3), current smoker (5.2; 2.7–10.1), obese (BMI 41 vs 26kg/m^2^: 1.4; 1–1.9), and report Māori (1.8; 1.2–2.8) or Pacific (1.6; 1.1–2.5) ancestry, compared to Caucasian. People with gout were more likely to report heavy SSB intake compared to people with diabetes (OR 2.4, 95% CI 1.5–3.9). Heavy SSB consumption reported by people with diabetes was similar if they did or did not require dialysis.

**Conclusion:**

A high proportion of patients with gout and type 2 diabetes, including those on haemodialysis, are not responding to health messages to abstain from SSB consumption.

## Introduction

Strong evidence links sugar sweetened beverage (SSB) consumption with weight gain [1], type 2 diabetes [2], gout [3,4], and increased risk of heart disease [5,6]. The relationship with weight gain is thought to be due to SSB contributing to a high glycemic load with lower satiety properties compared with energy derived from solid foods [7]. Several short term clinical trials have demonstrated the metabolic consequences of SSB intake such as an increase in inflammation [8], impaired beta-cell function and insulin sensitivity [9,10], and high blood pressure as well as accumulation of visceral adiposity and atherogenic dyslipidemia [11]. Biochemically, hepatic processing of sugar ingested in liquid form generates a rapid rise in urate [12–14], and there is epidemiological evidence that SSB interfere with the excretion of uric acid [3]. The relative risk of gout incidence was reported to be approximately two fold higher among those consuming two or more SSB per day, in two large prospective studies conducted in men [4] and women [15]. The risk of prevalent gout was reported to be up to 7-fold higher in Europeans drinking four or more SSB per day [3]. A meta-analysis of studies reporting the association between SSB consumption and risk of type 2 diabetes found that higher consumption of 1–2 servings per day was linked with 26% greater risk of developing diabetes than those drinking fewer than 1 serving per month [2].

Current American Diabetes Association and American College of Rheumatology guidelines recommend that individuals with type 2 diabetes and gout, respectively, limit their intake of drinks with added sugar [16,17]. Other lifestyle advice common to both diseases include smoking cessation, exercise to achieve physical fitness and weight loss for obese patients. Our aim was to determine sugar sweetened beverage intake among patients with gout or type 2 diabetes, and to describe the characteristics of those reporting high intake. We specifically included patients with type 2 diabetes who were receiving hemodialysis. These patients have regular dietitian input, both before initiation of renal replacement therapy and at least six monthly once on dialysis, with advice to limit fruit juice (due to potassium), dark colas (due to phosphate) and to restrict fluid intake, in addition to sugar restriction as part of diabetes management.

## Methods

### Participants

Participants aged 16 years or older were recruited from the Auckland and Christchurch areas in New Zealand, during 2009 to 2012, for a study of risk factors for gout and type 2 diabetes [18]. Information at study entry included age, sex, employment, smoking history, years of education completed, medical history, self-reported dietary and lifestyle factors and measurement of blood pressure, height and weight by trained assessors. All participants gave their written informed consent. No additional consent from next of kin, caretakers or guardians were obtained on behalf of participants aged 16–18 years as this is only required for those below the age of 16 years in New Zealand [19]. This study and written consent process was approved by the Multiregional Ethics Committee (MEC05/10/13).

### Data collection

Assessment of participants was undertaken by an interviewer-administered questionnaire in order to prevent literacy barriers preventing subjects taking part in this study. Participants were asked the ancestry of each of their grandparents. The Māori group comprised individuals who self-reported one or more Māori grandparent, the Pacific group included those who self-reported one or more Pacific grandparent (any with Māori grandparents were included in the Māori group) and the European Caucasian group consisted of people self-reporting four European Caucasian grandparents. Gout was ascertained by the 1977 American Rheumatism Association preliminary classification criteria [20]. Type 2 diabetes was ascertained by recorded physician-diagnosis, or the use of glucose lowering therapy. Education level was coded as years of school, college, and university completed using a scale of 1–20. Smoking history was coded as current smoker, ex-smoker or non-smoker. Dietary SSB intake was recorded as a circled number 0, 1, 2, 3, 4, 5, or more than 5 (as either a can or large glass of 300mL), in response to the question “how many sugar sweetened drinks (including fruit juice) but not including diet drinks, do you normally drink per day?” Fruit intake was recorded as a circled number 0, 1, 2, 3, 4, 5 or > 5 in response to the question “how many pieces of whole fresh fruit do you usually eat per day?” Years of education completed was recorded as a circled number from 1–20. Exercise habits was recorded as either sedentary, moderately active (defined as minor strenuous exercise at least 4 hours per week e.g.: walking or cycling), regular strenuous exercise (at least 3 hours per week e.g.: sports), or regular hard physical training (e.g.: for competition). BMI was calculated as weight in kilograms, divided by the square of height measured in metres.

### Statistical analysis

Data were checked for normality. SSB intake, reported initially as a continuous variable, was divided into three categories for analysis: zero, one to three and four or greater cans or large glasses per day. Individuals were divided by their disease status into three groups: gout (without diabetes), diabetes (with or without gout, but not receiving haemodialysis) and diabetes with haemodialysis treatment. The four categories of exercise habits was coded as 1 for sedentary, 2 for moderately active, 3 for regular strenuous exercise and 4 for regular hard physical training. Statistical analysis centred on considering evidence for different intake of SSBs by disease status, and how clinical and sociodemographic characteristics varied when high users of SSBs (4 or greater) were compared to those who reported three or less units per day. For continuous variables, the distribution was examined and if approximately normally distributed, Student’s t-test was used, otherwise, a non-parametric test was carried out (either the Wilcoxon rank-sum test for two comparisons or the Kruskal Wallis test for more than two categories). For categorical variables, chi-square tests were used. The threshold for statistical significance was five per cent and all tests were two tailed. Complete case analysis was used when data was missing for univariate analysis.

An exploratory logistic regression analysis was undertaken to consider the magnitude of association between various characteristics, and drinking four or more SSBs per day (compared to three or fewer), once other clinical factors had been accounted for. Age at assessment, systolic blood pressure, ethnic group, body mass index, smoking status and diagnosis group (gout, diabetes or diabetes on dialysis) were included in the multivariate model. Because many covariates were missing, we used multiple imputation. We used 10 imputations, so that the modelling results reported are based on averaging model parameters over 10 random imputations. Variables that were used in the multiple imputation modelling were: gender, age-at-enrolment, systolic blood pressure, smoking status, diagnosis group, ethnic group, body mass index and SSB intake. For continuous variables (age, BMI and systolic blood pressure), odds ratios were calculated at the 84^th^ and 16^th^ centiles of the distribution, since this approximates the comparison for a binary variable [21].

All analyses were done using R software (version 3.1.1). The package *rms* (Regression Modeling strategies 3.3–2 version 2011) was used for the logistic regression analysis and the functions *aregImpute* and *fit*.*mult*.*impute* for multiple imputation. The *aregImpute* function finds transformations that optimise how each variable may be predicted from every other variable, using additive semiparametric models. *fit*.*mult*.*impute* was then used to average sets of regression coefficients and compute variance and covariance, adjusted for the error derived from the uncertainty from imputation of missing data.

## Results

A total of 1023 people with gout and 580 people with type 2 diabetes, of whom 206 were receiving hemodialysis, provided data for analysis (Table 1). Data on daily SSB intake and fruit intake was available on 96% (1539 and 1535/1603 respectively) of participants. BMI data was available on 98% (1575/1603) of participants, waist measurements on 89% (1434/1603) and blood pressure on 74% (1193/1603). Years of education was recorded in 72% of participants (1161/1603). Missing data was greatest for smoking status (713/1603) and exercise levels (727/1603) which was only reported by 44% and 45% of participants, respectively.

**Table 1:** Characteristics of subjects by disease category.

	Diabetes without dialysis	Diabetes with dialysis	Gout without diabetes or dialysis	Test stat.	*P* value
**Total**	374	206	1023		
**Age years (n = 1603)** Median (IQR)	57 (49,64)	57 (47,63)	52 (43,61)	Kruskal-Wallis test	<0.001
**Sex (n = 1603)**				Chi square	<0.001
**Male n (%)**	250 (66.8)	97 (47.1)	911 (89.4)		
**Female n (%)**	124 (33.2)	109 (52.9)	108 (10.6)		
**Ethnicity (n = 1603)**				Chi square	<0.001
**Caucasian n (%)**	71 (19.1)	72 (35.1)	405 (39.7)		
**Māori n (%)**	124 (33.3)	66 (32.2)	201 (19.7)		
**Pacific n (%)**	150 (40.3)	50 (24.4)	332 (32.6)		
**Other n (%)**	27 (7.3)	17 (8.3)	81 (8.0)		
**Education years (n = 1161)** Median (IQR)	11 (10,13)	11 (11,13)	12 (11,15)	Kruskal-Wallis test	0.0038
Exercise* (n = 727) Median (IQR)	2 (1,2)	2 (1,2)	2 (2,3)	Kruskal-Wallis test	<0.001
**SSB consumption (n = 1539)** Median (IQR)	0.15 (0,2)	0.5 (0,2)	1 (0,3)	Kruskal-Wallis test	<0.001
**0 SSB per day n (%)**	186 (51.4)	107 (52.7)	352 (36.1)	Chi square	<0.001
**≥1 SSB per day n (%)**	176 (48.6)	96 (47.3)	622 (63.9)		
**<4 SSB per day n (%)**	331 (91.4)	185 (91.1)	795 (81.6)	Chi square	<0.001
**≥4 SSB per day n (%)**	31 (8.6)	18 (8.9)	179 (18.4)		
**Daily Fruit intake(n = 1535)** Median (IQR)	2 (1,3)	1 (0.5,2)	2 (1,3)	Kruskal-Wallis test	<0.001
**Smoking status (n = 713)**				Chi square	0.0034
**Non-Smoker n (%)**	89 (48.4)	69 (35.4)	175 (52.4)		
**Ex-smoker n (%)**	74 (40.2)	105 (53.9)	129 (38.6)		
**Smoker n (%)**	21 (11.4)	21 (10.8)	30 (9.0)		
**BMI (kg/m** ^**2**^ **) (n = 1575)** Median (IQR)	35.4 (31.2,41.4)	27.9 (24.3,33.7)	31.9 (28.4,36.6)	Kruskal-Wallis test	<0.001
**Waist (cm) (n = 1434)** Median (IQR)	115 (104,127)	98 (91,113)	107 (98,117)	Kruskal-Wallis test	<0.001
**Blood pressure (n = 1193)**					
**Systolic BP (mmHg)** Median (IQR)	132 (122,146)	139 (121,157)	135 (122,148)	Kruskal-Wallis test	0.1876
**Diastolic BP (mmHg) Median (IQR)**	81 (75,90)	73 (61,83)	81 (74,90)	Kruskal-Wallis test	<0.001

* Exercise category was defined as 1 for sedentary, 2 for moderately active, 3 for regular strenuous exercise and 4 for regular hard physical training.

Adherence with abstinence from SSB consumption was lowest (36%, 352/974) among those who had gout, 53% (107/203) among those with type 2 diabetes requiring hemodialysis, and 51% (186/362) among those with type 2 diabetes, not requiring hemodialysis. Fruit intake was similar among non-dialysis patients with gout or type 2 diabetes, but was lower among patients receiving hemodialysis. Median reported exercise was higher among those with gout. Median BMI was highest among those with type 2 diabetes without hemodialysis, followed by those with gout and was lowest among those receiving hemodialysis. This pattern was also seen for neck and waist measurements. However, blood pressure was similar among all three groups. Current smoking was reported by 9% (30/334) of those with gout and 11% of those with type 2 diabetes, with (21/195) or without hemodialysis (21/184).

High intake (4 or more SSBs per day) was reported by 18% (179/974) of those with gout and 9% of those with diabetes, with (18/203) or without (31/362) hemodialysis. The characteristics of these heavy SSB consumers are shown in Table 2, and include lower age, greater BMI, male gender, Māori or Pacific ancestry, gout, and higher current smoking status. The median BMI was significantly higher among those consuming 4 or more SSBs per day than those consuming less (34 vs 32 kg/m2, p<0.001). Blood pressure was not significantly different among heavy and low SSB consumers. The multivariable analysis (Table 3) showed that factors associated with heavy SSB consumption (4 or more drinks per day, compared to fewer) included current smoking, male gender, younger age, higher BMI, Māori or Pacific ethnicity (relative to Caucasian), and gout (relative to diabetes status). The greatest association with heavy SSB intake was with current smoking status, compared to non-smokers (adjusted odds ratio 5.2, 95% CI: 2.7–10.1), however those of male gender (adjusted odds ratio 1.8, 95% CI: 1.1 to 2.9) and younger age (40 years compared to 65; adjusted odds ratio 1.6, 95% CI 1.1 to 2.3) were also more likely to report heavy SSB intake. Patients with gout were more likely to report heavy SSB intake compared to those with diabetes without dialysis (adjusted odds ratio 2.4, 95% CI 1.5–3.9). There was no significant difference in SSB intake among those with diabetes who were on dialysis compared to those who were not.

**Table 2:** Characteristics of heavy SSB consumers.

	Consuming <4 SSB per day	Consuming ≥4 SSB per day		
Total	1311	228	Test stat.	P value
**Age years** median(IQR)	55 (46,63)	48 (40,58)	Rank-sum test	<0.001
**Male n (%)**	1001 (77.0)	199 (87.0)	Chi square	< 0.001
**Female n (%)**	306 (23.0)	29 (13.0)		
**Caucasian n (%)**	472 (36.2)	52 (22.8)	Chi square	< 0.001
**Māori n (%)**	308 (23.6)	70 (30.7)		
**Pacific n (%)**	409 (31.4)	99 (43.4)		
**Other n (%)**	115 (8.8)	7 (3.0)		
**Education (years)** median (IQR)	12 (11,15)	11 (11,13)	Rank-sum test	0.0087
Exercise category* Median (IQR)	2 (1,2)	2 (1,2)	Rank-sum test	0.0668
**Daily fruit intake** Median (IQR)	2.0 (1.0,3.0)	1.5 (1.0,2.5)	Rank-sum test	0.1745
**Non smoker n (%)**	312 (49.0)	18 (27.7)	Chi square	< 0.001
**Ex-smoker n (%)**	273 (42.9)	29 (44.6)		
**Smoker n (%)**	52 (8.2)	18 (27.7)		
**BMI (kg/m** ^**2**^ **)** Median (IQR)	32.0 (28.0–37.2)	34.0 (29.8–40.5)	Rank-sum test	<0.001
**Waist** (cm) Median (IQR)	108 (98,119)	112 (102,126)	Rank-sum test	< 0.001
**Neck (cm)** Median (IQR)	42 (39,45)	42 (40,46)	Rank-sum test	0.4095
**Systolic BP (mmHg)**	134 (122,148)	135 (123, 148)	Rank-sum test	0.8981
**Diastolic BP (mmHg)**	80 (73, 90)	80 (74, 90)	Rank-sum test	0.7064

* Exercise category was defined as 1 for sedentary, 2 for moderately active, 3 for regular strenuous exercise and 4 for regular hard physical training.

**Table 3:** Multivariable associations with heavy SSB consumption patterns.

Risk factor	Reference	Comparison	Crude odds ratio (95% CI)	*Adjusted odds ratio (95% CI)
**Gender**	Female	Male	2.1 (1.4–3.2)	1.8 (1.1–2.9)
**Age (years)**	65	40	2.7 (2.0–3.6)	1.6 (1.1–2.3)
**Ethnic group**	Caucasian	Māori	2.1 (1.4–3.0)	1.8 (1.2–2.8)
	Caucasian	Pacific	2.2 (1.5–3.1)	1.6 (1.1–2.5)
**Smoking status**	Non-smoker	Smoker	5.9 (3.2–10.8)	5.2 (2.7–10.1)
	Non-smoker	Ex-smoker	1.6 (0.9–2.6)	1.6 (0.9–2.7)
**Disease**	Diabetes—no dialysis	Gout	2.3 (1.6–3.5)	2.4 (1.5–3.9)
	Diabetes—no dialysis	Diabetes with dialysis	1.0 (0.6–1.8)	1.3 (0.7–2.6)
**Body mass index** ^†^ **(kg/m** ^**2**^ **)**	26.4	40.8	1.6 (1.3–2.0)	1.4 (1.0–1.9)
**Systolic blood pressure** ^†^	118	156	1.0 (0.8–1.3)	1.0 (0.7–1.3)

*Adjusted for all other variables present in table

^†^For continuous variables comparisons were taken at the 16^th^ and 84^th^ centiles.

## Discussion

This study is based on the assumption that people with gout or type 2 diabetes (particularly those on dialysis) should not be drinking SSB due to the detrimental health effects of excess dietary sugars on obesity, glycemic control, gout and myocardial infarction [3,4,15,22,23].

We found a high prevalence of daily consumption of at least one SSB among those with gout (63.9%) and type 2 diabetes, both by those who were on hemodialysis and those who were not (47.3% and 48.6%). Of concern, 18% of patients with gout, and 9% of patients with type 2 diabetes, (including those receiving hemodialysis treatment), reported drinking more than 4 SSBs per day. This compares to a prevalence of daily consumption of SSB among the general New Zealand population of 14.3% from the 2008/2009 New Zealand adult nutrition survey [24]. The daily consumption of 1 SSB exceeds the recommended sugar intake for those affected with diseases such as gout and type 2 diabetes, due to the detrimental health outcomes of excess dietary sugars on obesity, glycemic control, and risk of ischaemic heart disease.

The general guidelines for intake of added sugar as recommended by the American Heart Association is no more than 5 teaspoons per day (20g) for an average woman and 9 teaspoons (36g) per day for an average adult man [25]. Given that one regular can of SSB contains about 8 teaspoons (32g), of added sugar, this is the limit of intake recommended for the general population, while those with gout and type 2 diabetes are advised to abstain completely from SSB intake. This is due to the detrimental effects of excess dietary sugars on blood pressure, lipids, inflammation, gout, type 2 diabetes and risk of myocardial infarction [3,4,15,22,23]. High intakes of SSB of around 4 servings per day (1L) have been shown to significantly increase liver fat and triglycerides over a 6 month study period [26]. Furthermore, the association of one SSB with a 20% higher risk of myocardial infarction, from cohort studies, provides compelling evidence to reduce intake [5] [6]. Replacing SSB consumption with water or other low energy drinks can offer a relatively simple, low-cost option to improve gout and glycemic control in addition to achieving weight control and improving cardiovascular risk.

Those who consume 4 or more SSBs per day are ingesting approximately 520 calories per day in liquid form, so it is not surprising that they also had higher BMI measurements (34 vs 32 kg/m^2^, p <0.001). The association of increased SSB intake with increased BMI has been reported in recent systematic reviews in other populations [27,28]. Other characteristics of heavy SSB consumers in the multivariable analysis included male gender, lower age, Pacific or Māori ancestry, and those who were current smokers. Those who had gout were more likely to report heavy intake compared to those who had diabetes. Blood pressure was similar between different groups of high and low SSB intake, suggesting similar adherence to prescribed antihypertensive medications.

These results suggest enhanced strategies for reducing or eliminating SSB intake need to be devised particularly targeting younger, overweight, adult male smokers of Māori or Pacific ancestry with gout and diabetes. Practical opportunities to reduce SSB intake among patients with gout, type 2 diabetes, including those on hemodialysis (who are at higher risk of cardiovascular mortality), may be to ensure SSB intake is routinely assessed and recorded in clinical practice, similar to blood pressure monitoring and smoking status, particularly among younger, overweight males of Māori or Pacific ancestry. Those patients declaring SSB intake may then be given brief advice to stop, with low sugar alternatives discussed.

The impact of ethnic and cultural factors driving SSB intake has been reported among the United States population with higher proportions of American Indian [29] and African-Americans [30,31] consuming SSBs, and higher frequency among younger people and those with fewer years of formal education [29]. Lowest SSB consumption has been reported among Asian Americans [32], including Chinese-American [33] and Korean-Americans [34], compared to white Americans [35].

Our results, among patients with type 2 diabetes, are similar to a US study which reported 45% of adults with diabetes consuming one or more SSB per day, with higher intakes reported among men, young adults, non-Hispanic black and lower income individuals [36]. However, there are very few reports documenting intake of SSBs among people with renal failure or gout.

A surprising feature of our study was the relatively high intake of SSBs in those treated with hemodialysis as a complication of diabetes (8% reported intake of four or more SSBs per day similar). SSB intake among this group of patients has not previously been reported. High SSB intake among people with diabetes was similar whether or not they were treated with dialysis, in the multivariable analysis. Possible explanations for this finding may include lack of adherence to health messages in general, reflected in those who had end-stage renal complications of diabetes, since 11% of this group also smoked. Alternatively, these patients may have a sugar addiction syndrome, driving continued excessive intake to relieve unpleasant withdrawal symptoms, which has been described in animal studies [37].

Given that non-adherence with advice to limit SSB intake was greater among patients with gout than with type 2 diabetes, this suggests that the intensity and frequency of this message given to those affected by gout may be less than that given to those with type 2 diabetes, who are more likely to be provided with dietician support. The relatively recent inclusion of SSB avoidance in the gout management guidelines [17], as compared with long-standing SSB avoidance messages in type 2 diabetes guidelines, is a likely explanation. Alternatively, the link between high blood glucose measurements and intake of sugar may be understood better by people with type 2 diabetes. Nonetheless, almost half of patients with type 2 diabetes still reported drinking at least one or more SSB per day and almost one in ten reported drinking four or more SSBs per day.

Limitations of this study include its cross-sectional design, which allows identification of association but not causality. There is a possibility of social desirability influencing reporting of SSB consumption, however this is unlikely to have introduced a systematic bias in this study. Most studies suggest that under-reporting is more common than over-reporting, which should result in under-estimation of the actual SSB intake among such high risk populations [28]. We acknowledge limitations of missing data, particularly for some variables, such as smoking and blood pressure. The reason for this is largely due to the iterative process by which the data collection forms were refined to include additional clinical characteristics over the duration of the study. Earlier participants were less likely to have complete data recorded than later ones. It is, therefore, unlikely that systematic bias occurs in people with missing data, and the missing at random assumption of our multivariate analysis, and multiple imputation is likely to be valid.

## Conclusion

This study detected a high prevalence of SSB intake among adult New Zealanders with gout and type 2 diabetes, including people treated with hemodialysis. Risk factors for high intake included younger age, male gender, higher BMI, Pacific or Māori ancestry and current smoking. Awareness of this pattern by physicians and health professionals involved in the care of these patients is important to address, since limiting intake is likely to reduce complications of the disease.
